# Physical Activity and Brain Function in Older Adults at Increased Risk for Alzheimer’s Disease

**DOI:** 10.3390/brainsci3010054

**Published:** 2013-01-14

**Authors:** J. Carson Smith, Kristy A. Nielson, John L. Woodard, Michael Seidenberg, Stephen M. Rao

**Affiliations:** 1 Department of Kinesiology, School of Public Health, University of Maryland, College Park, MD 20742, USA; 2 Department of Psychology, Marquette University, PO Box 1881, Milwaukee, WI 53201, USA; E-Mail: kristy.nielson@marquette.edu; 3 Department of Neurology, Medical College of Wisconsin, 8701 Watertown Plank Road, Milwaukee, WI 53226, USA; 4 Department of Psychology, Wayne State University, 5057 Woodward Ave, Detroit, MI 48202, USA; E-Mail: john.woodard@wayne.edu; 5 Department of Psychology, Rosalind Franklin University of Medicine and Science, 3333 Green Bay Rd, North Chicago, IL 60064, USA; E-Mail: michael.seidenberg@rosalindfranklin.edu; 6 Schey Center for Cognitive Neuroimaging, Neurological Institute, Cleveland Clinic, 9500 Euclid Ave/U10, Cleveland, OH 44195, USA; E-Mail: raos2@ccf.org

**Keywords:** Alzheimer’s disease, cognition, exercise, physical activity, APOE genotype, genetic risk, mild cognitive impairment, memory, MRI, neuroimaging

## Abstract

Leisure-time physical activity (PA) and exercise training are known to help maintain cognitive function in healthy older adults. However, relatively little is known about the effects of PA on cognitive function or brain function in those at increased risk for Alzheimer’s disease through the presence of the apolipoproteinE epsilon4 (APOE-ε4) allele, diagnosis of mild cognitive impairment (MCI), or the presence of metabolic disease. Here, we examine the question of whether PA and exercise interventions may differentially impact cognitive trajectory, clinical outcomes, and brain structure and function among individuals at the greatest risk for AD. The literature suggests that the protective effects of PA on risk for future dementia appear to be larger in those at increased genetic risk for AD. Exercise training is also effective at helping to promote stable cognitive function in MCI patients, and greater cardiorespiratory fitness is associated with greater brain volume in early-stage AD patients. In APOE-ε4 allele carriers compared to non-carriers, greater levels of PA may be more effective in reducing amyloid burden and are associated with greater activation of semantic memory-related neural circuits. A greater research emphasis should be placed on randomized clinical trials for exercise, with clinical, behavioral, and neuroimaging outcomes in people at increased risk for AD.

## 1. Introduction

Cognitive decline in late life is associated with loss of independence, functional decline in activities of daily living, nursing home placement, and mortality [1,2,3]. The prevalence of cognitive impairment among older adults in the U.S. is estimated to be over 20 percent and, with an aging U.S. population, cognitive impairment and Alzheimer’s disease (AD) constitutes an alarming public health problem [4]. Despite the known risk factors and primary neuropathology of AD, a multidisciplinary panel of experts recently concluded that there is insufficient evidence to support the use of pharmaceutical agents or dietary supplements to prevent cognitive decline or AD [5]. Efforts are underway to identify preclinical biomarkers in order to predict future cognitive decline and AD more effectively [6,7,8,9]. The early identification of vulnerable individuals may permit targeted early intervention and prevention trials. Interventions, including modifiable lifestyle behaviors [10], that even modestly delay the onset of cognitive impairment or help maintain cognitive function in older adults may slow the development of AD and will have a major public health impact. A physically active lifestyle and greater cardiorespiratory fitness have long been associated with reduced all-cause mortality [11,12,13], and more recently with reduced mortality attributable to dementia [14,15]. Moreover, in both healthy human subjects [16] and in animal models [17,18], exercise and PA have been shown to benefit cognitive function and may offer neuroprotection. Leisure-time PA, greater cardiorespiratory fitness, and aerobic exercise training have been associated with better cognitive function in healthy older adults, especially on tasks sensitive to age-related memory decline [19,20,21]. In animals, the neuroprotective effects of exercise have been shown to be particularly robust in the hippocampal complex and dentate gyrus [16,22,23]. These brain areas are important to learning and memory and are attacked early in the course of AD, the most common cause of dementia in older adults [24]. 

An NIH consensus panel on Alzheimer’s disease, while noting the encouraging preliminary evidence for PA and exercise training to help mitigate cognitive decline and AD, concluded that there is no solid evidence that any modifiable factor, including PA, is associated with reduced risk for AD [5]. Most of the investigations considered by the NIH consensus panel were not large randomized clinical trials, and most did not consider the genetic risk status of the study participants, or specifically excluded individuals with mild memory loss or metabolic disease that increase the risk of cognitive decline. While many epidemiological studies have shown that leisure-time PA may be a lifestyle behavior that reduces the risk of incident cognitive impairment [25,26] and AD [27], this effect has not been consistently reported (see [28] for review). In a recent meta-analysis of prospective studies, Sofi and colleagues [29] reported that cognitively intact older adults who reported engaging in high levels of PA at baseline had a 38% reduced risk (hazard ratio (HR) 0.62, 95% confidence interval (CI) 0.54–0.70) for incident cognitive decline at follow-up (range 1–12 years). A similar effect was observed for those who reported low to moderate amounts of PA compared to sedentary individuals (HR 0.65, 95% CI 0.57–0.75). Based on the average effects among these prospective studies, PA appears to play an important role in maintaining cognitive function in healthy older adults. However, until recently there has been little attention paid to whether or not PA is more or less effective at preserving cognitive function or brain function in those at increased risk for AD. Differential effects of exercise on cognitive status or brain function that depend on AD-risk status (e.g., greater effects in those at greater risk for AD) may account for a portion of unexplained variance in previous studies and help explain disparate findings, and could have important clinical implications. Here, we propose that the question of whether PA and exercise interventions may impact future cognitive decline and brain function, and possibly slow AD progression, may best be addressed by focusing on those individuals at greatest risk for AD. 

## 2. Risk for AD

The neuropathological changes associated with AD may occur decades prior to the clinical diagnosis [30]. There are several factors that may play a role in the development of AD-related neuropathology, and additionally, whether or not a subsequent clinical diagnosis of AD is realized in the face of such neuropathology. The most consistently reported factors are the presence of the apolipoprotein-E (APOE) ε4 allele, a diagnosis of mild cognitive impairment (MCI), and impaired glucose tolerance and type 2 diabetes mellitus [31,32,33]. Although advanced age is associated with greater probability of incident AD and is considered a risk factor [4] (but see also [34]), this review will be restricted to studies of older adults and as such will not consider effects of PA on brain function that vary across the aging spectrum. 

The APOE ε4 allele on chromosome 19 is associated with a higher risk and decreased age of onset of AD, whereas the presence of ε2 may be protective against AD [33]. The ε4 allele is associated with several biochemical processes that may be implicated in the etiology of AD (*i.e.*, amyloid deposits, neurofibrillary tangle formation, neuronal cell death, oxidative stress, synaptic plasticity, and cholinergic signaling dysfunction). However, AD can develop in the absence of the ε4 allele [35] and a large proportion of ε4 allele carriers never develop the disease [36]. 

The preclinical stage of AD may be divided into two periods: a “latent” phase with no observable symptoms and a “prodromal” phase of mild symptoms that do not meet diagnostic criteria for probable or possible AD. The prodromal phase of AD is characterized by progressive isolated memory deficits, a condition termed mild cognitive impairment (MCI) [37]. Amnestic MCI criteria include subjective and objective demonstration of episodic memory impairment without generalized dementia and with intact activities of daily living [38]. This subtype represents a high risk state for AD, with 40% progressing to AD over a 4-year period [39] and approximately 60% exhibiting autopsy-verified AD post-mortem [40]. More recently, a panel of experts made recommendations to revise the criteria for the diagnosis of MCI and dementia along a spectrum of pre-clinical to clinical symptom presentation coupled with biomarkers for AD-related pathology [41,42]. Currently, there is a need to find effective therapies that can enhance cognitive function of patients diagnosed with MCI. 

Additionally, recent evidence suggests that individuals diagnosed with type 2 diabetes mellitus are at greater risk for the development of AD [43], independent of vascular risk factors [44]. In addition, both diabetes and impaired fasting blood glucose increase the risk of mild cognitive impairment (MCI), which predicts increased risk for dementia [45]. In a quantitative review of 25 prospective studies, Cukierman *et al*. [46] found that individuals with diabetes were at 1.6 times (95% CI 1.4–1.8) greater risk for all-cause dementia compared to older adults without diabetes.

In addition to APOE genotype, MCI diagnosis, and type 2 diabetes mellitus, there are several other known risk factors for AD including major depression, hypertension, cardiovascular disease, and obesity [31,47,48]. However, there is insufficient evidence to evaluate the interactions between these conditions and PA behavior, cardiorespiratory fitness, or exercise training on cognitive or brain imaging outcomes.

## 3. Physical Activity, Cognitive Function, and Increased Risk for AD

Two studies have concluded that the protective effects of PA on cognitive function and the incidence of cognitive impairment are larger in those who possess a genetic risk as a carrier of the APOE-ε4 allele [49,50]. Schuit and colleagues were the first to report an interaction between PA and APOE genotype on cognitive decline. They followed 560 Dutch men over three years. Physical activity was assessed at baseline through self-report and cognitive decline was defined as a decrease of three or more points on the Mini-Mental State Examination (MMSE; [51]) at the three-year follow-up assessment. The odds ratio for cognitive decline was not associated with PA status in APOE-ε4 allele non-carriers (who had overall lower risk). However, among APOE-ε4 allele carriers, those who were physically active less than one hour per day had an odds ratio for cognitive decline that was nearly 4 times greater than APOE-ε4 allele carriers who reported more than one hour per day of PA. This association was significant after adjusting for age, education, and baseline MMSE score.

Rovio and colleagues examined 1449 older adults (ages 65–79) from a longitudinal population-based survey over an average follow-up period of 21 years [52]. Participants were classified as physically active or sedentary based on their self-reported PA at midlife as determined from responses to the question, “How often do you participate in leisure-time PA that lasts at least 20–30 minutes and causes breathlessness and sweating?” ([52], p. 704). Those who reported two or more days per week were classified as physically active and those who reported less than two days per week were classified as sedentary. There were no differences between the active and sedentary groups in the number of APOE-ε4 allele carriers, and the analysis of risk for AD was adjusted for age at follow-up, sex, education, follow-up time, locomotor dysfunction, midlife body mass index, systolic blood pressure, cholesterol, history of myocardial infarction, stroke, and diabetes mellitus. Compared to the sedentary group, the odds ratio for AD at follow-up was 0.34 (95% CI 0.25–0.77) in the physically active group. Furthermore, the effect of being physically active was found to be stronger in APOE-ε4 allele carriers (OR 0.23, 95% CI 0.07–0.74) compared to non-carriers (OR 0.59, 95% CI 0.21–1.69). This interaction between PA and APOE allele status was similar after further adjustment for smoking status and alcohol consumption. 

Lindsay and colleagues reported the results of the Canadian Study on Health and Aging, a prospective study of 4615 who were followed for 5 years [53]. Physical activity was assessed with a single item self-report question (among a large survey) that asked if the participant engaged in regular PA (yes/no); however, the term “regular” was not defined for the participant. Possession of an APOE-ε4 allele, compared to APOE-e3 allele homozygotes, resulted in a 3.28 fold increased risk for AD (95% CI 1.98–5.44), whereas being regularly physically active (*vs.* not regularly physically active) reduced the risk for AD (OR 0.69, 95% CI 0.50–0.96). However, the effects for PA were not modified by APOE allele status, age, or sex. 

Finally, one observational study reported stronger effects of PA in APOE-ε4 non-carriers. Podewils and colleagues analyzed data from 3075 participants in the Cardiovascular Health and Cognition Study [54]. Self-reported PA over the past 2-weeks, expressed as kcal/week and also as the total number of activities performed over 2 weeks (range 0–14 total activities), was aggregated from two baseline assessments in 1989–1990 and 1992–1994. Incident dementia (all-cause, AD, and vascular dementia) was determined in 1999–2000 after an average 5.4-year follow-up (480 all-cause dementia cases). There were no differences in the incidence of all-cause dementia in the highest quartile of weekly PA energy expenditure (> 1,657 kcal/week) compared to the referent quartile (<248 kcal/week) when adjusted for age (HR 0.82, 95% CI 0.64–1.07) or multiple additional factors (HR 0.85, 95% CI 0.61–1.19). A similar pattern was observed when the analysis was restricted to incidence of AD (age-adjusted HR 0.71, 95% CI 0.49–1.03; adjusted HR 0.70, 95% CI 0.44–1.13) and vascular dementia. However, larger effects were observed when PA was expressed as the total number of different types of physical activities performed. The incidence of all-cause dementia and AD were found to be significantly reduced in those who reported engaging in four or more physical activities compared to the referent group who reported zero physical activities over the past 2-weeks (adjusted HRs 0.58, 95% CI 0.41–0.83 and 0.55, 95% CI 0.34–0.88, respectively). For vascular dementia the incidence was reduced in those with four or more activities when adjusted for age (HR 0.59, 95% CI 0.39–0.90), but not when fully adjusted (HR 0.59, 95% CI 0.39–1.08). 

When these effects were stratified by APOE allele status, there was not a significant reduction in incidence of all-cause dementia for any quartile of energy expenditure among APOE-ε4 carriers and non-carriers. The only significant interaction for all-cause dementia occurred when PA was expressed in number of activities. APOE-ε4 non-carriers who reported four or more activities showed a lower incidence (adjusted HR 0.44, 95% CI 0.28–0.69) but APOE-ε4 carriers did not (adjusted HR 1.20, 95% CI 0.63–2.29). A possible explanation for the inconsistency in the interaction effects when expressed as energy expenditure *versus* number of activities is that there were a significantly greater proportion of ε4 carriers (who, as a whole, showed greater odds for incident dementia) in the most physically active energy expenditure group compared to the referent group, potentially biasing the results, which was not the case for the most active group performing four or more activities. In addition, aggregate baseline PA measured from assessments obtained 3–5 years apart raises the question of whether the PA patterns were stable, or perhaps increased or decreased differentially over time in the quartile groups. Collectively, these aspects of the Podewils *et al*. study, in addition to differences across studies in the measure of PA, may explain why they did not replicate the effects of PA in APOE-ε4 carriers that have been reported by others [50,52].

Etnier *et al*. [49] measured cardiorespiratory fitness using indirect calorimetry (measured from a maximal graded exercise test) in 94 women aged 51 to 81 (mean age = 62 years). They found that greater cardiorespiratory fitness was associated with better performance on the Rey Auditory Verbal Learning Test (Trial 5 performance) [55], the Rey-Osterreith Complex Figure Test [56], and Paced Auditory Serial Addition Task [57] only in healthy older women who were APOE-ε4 homozygotes, but not APOE-ε4 heterozygotes or non-carriers. The interaction effects were based on only eight APOE-ε4 homozygotes, so these effects need to be replicated with a larger sample to rule out a spurious or sample specific effect. Also, this study did not have a prospective design, did not assess clinical outcomes, and included fewer subjects than the population based studies described above. However, it is noteworthy for its rigorous characterization of cardiorespiratory fitness and use of a battery of standardized neuropsychological outcomes.

In summary, observational studies have reported inconsistent effects of PA on cognition in those at greater genetic risk for AD. In two studies, greater effects of PA on cognition or future incidence of cognitive decline have been observed in those who carry at least one copy of the APOE-ε4 allele. Despite one study that showed greater effects of fitness in APOE-ε4 homozygotes, the dose response effects of APOE-ε4 genotype, or the combined and comparative effects of the less common and protective ε2 genotype, have not been adequately addressed. The current inconsistencies in the literature may be best explained by differences in how PA was measured and the levels of PA that were used to define greater PA, which in some cases were not at a standard that meets current PA recommendations for older adults. These observational studies also vary in the timing of when the single estimate of PA behavior was assessed relative to the outcome of interest and little information is available regarding potential changes in PA behavior after the baseline assessment was obtained, which are concerns that are more easily addressed in intervention studies.

## 4. Exercise Training in Mild Cognitive Impairment

There have been several investigations regarding the effects of an exercise intervention in AD patients [58,59,60,61,62], which have been reviewed elsewhere [63]. Similarly, there are several studies that have assessed the effects of exercise training on cognition in healthy older adults without identifiable AD risk factors (e.g., [23,64]; for reviews see [19,20,65,66,67]). There are no prospective exercise intervention studies that have compared individuals based on genetic risk status. There have been several studies, however, that have examined the effects of exercise training in older adults at risk for AD due to their diagnosis of MCI. 

In the first reported clinical trial of an exercise intervention in older adults diagnosed with MCI, Scherder *et al*. [68] assigned 43 (38 women, mean age 86 years) “frail” residents of an assisted living/nursing home facility to 6 weeks (30 min per session, three sessions/week) of “self-paced slow walking with an aid” or hand and face exercises, or a usual care control condition. Cognitive tests were administered at baseline, immediately after the 6-week intervention and control conditions, and after a 6-week follow-up in the absence of any intervention. Individuals in both the walking and hand/face exercise conditions showed significantly improved scores for category naming (verbal fluency) after the 6-week intervention compared to the control group, but these scores did not differ from the control condition at the 6-week follow-up. There were also no significant effects of the exercise treatments on performance on Part A or Part B of the Trail-Making Test or on various tests of episodic and working memory. Thus, other than the short-lived benefits to verbal fluency, the exercise intervention failed to enhance other aspects of cognitive performance. A concern is that the exercise intervention was not well described and may not have been sufficient to result in improved fitness. Brief exercise programs (less than 12 weeks) that are not at least moderately intense (>60% VO_2max_) may not produce significant gains in cardiorespiratory fitness [69,70]. While gains in cardiorespiratory fitness are not required to improve multiple aspects of cognitive function [19,20,66], it is not clear that the exercise described by Schereder *et al*. [68] extended much beyond what would be typically experienced by a frail yet ambulatory resident of an assisted living or nursing home facility. The lack of effect on cognitive outcomes in this MCI sample is consistent with a recent review of exercise interventions in dementia patients which concluded that self-paced walking interventions conducted within a care facility had no effect on cognitive function [63].

Lautenschlager *et al*. conducted a randomized trial in 170 older adults (mean age 69 years) with subjective memory complaints (*n* = 59 single domain amnestic MCI, *n* = 28 multi-domain amnestic MCI; *n* = 15 non-amnestic MCI; *n* = 68 not clinically diagnosed) [71]. Participants were assigned to a home-based exercise program that asked participants to complete three 50-min unsupervised sessions per week (usually walking). The participants wore activity accelerometers for 7 consecutive days, and kept activity diaries, at baseline and 6, 12, and 18 months during the intervention. The total number of steps per week was reported. Among those who completed the study, participants in the exercise condition *increased* their stepping between approximately 3200 and 8900 steps per week over baseline, while the participants in the control condition *decreased* their stepping between approximately 500 and 4000 steps per week compared to their baseline. Those who were assigned to the home-based PA program showed significantly greater change scores on the Alzheimer Disease Assessment Scale-Cognitive (ADAS-Cog) subscale compared to the usual care condition. The difference between the groups was likely due to the deterioration in ADAS-Cog scores in the usual care group (change score 1.04 points, 95% CI 0.32–1.82) as the improved scores in the exercise group did not meet the threshold for significance (change score −0.26 points, 95% CI −0.89–0.54). Thus, it may be more appropriate to interpret the effect of exercise intervention as leading to cognitive stability relative to the usual care control condition, not necessarily improved cognition.

Miller and colleagues conducted a single-arm clinical trial in individuals diagnosed with MCI who lived in a single assisted living facility [72]. The 31 participants (mean age 84 years; mean education 15.7 years) all scored 0.5 on the Clinical Dementia Rating (CDR) Scale (indicating very mild or questionable cognitive impairment) and also received some level of assistance with activities of daily living. The 6-month intervention was performed in a group setting and consisted of low intensity exercise (marching in place, knee lifts) combined with resistance exercise and flexibility training two sessions per week, approximately 60 min per session. At baseline and after the 6-month intervention, a battery of neuropsychological tests was administered, blood was drawn for analysis of β-amyloid 1–38, β-amyloid 1–40 and β-amyloid 1–42, and fitness was estimated using a 2-min step test (number of steps). Participants did not improve on any of the neuropsychological measures of memory, executive function, or semantic fluency, and showed a significant decline in performance on the Trail Making Test, Part B and animal naming (verbal fluency) tasks. There was no effect of the intervention on blood β-amyloid levels. Although participants improved in their step test performance, there was no physiological (e.g., heart rate) or perceptual (e.g., rating of perceived exertion) measure to indicate the relative strain of the exercise. It is unlikely that the intensity and frequency of the exercise resulted in substantial changes in cardiorespiratory fitness (*i.e.*, VO_2peak_). Also, a control condition was not included so the independent effects of the passage of time cannot be determined. Due to these limitations, it is difficult to accurately interpret the effects reported by Miller *et al*. [72].

Baker *et al*. [73] conducted a six-month randomized clinical trial in 33 adults diagnosed with amnestic MCI. Twenty-three participants completed a high-intensity (75%–85% of heart rate reserve) aerobic exercise-training program, and 10 completed a stretching control condition (intensity < 50% heart rate reserve). Both groups completed four sessions per week that were 45–60 min in duration. The exercise program consisted of supervised exercise sessions for two weeks, then once weekly supervision thereafter, on a treadmill (most commonly selected mode), stationary bicycle, or elliptical trainer. Changes in cardiorespiratory fitness were documented using indirect calorimetry during a maximal graded exercise test. They found that exercise training resulted in significant improvement in women on the Symbol-Digit Modalities Test, and Verbal Fluency (category fluency), an index of semantic processing ability. Performance on the Stroop Color-Word Interference Task also improved in women, but not in men, in the exercise training condition relative to the stretching control condition. Other measures of cognitive function were not significantly affected by the exercise intervention. In regard to peripheral metabolic effects, women but not men in the exercise condition showed improved insulin sensitivity relative to the control condition. The results of this study provide evidence that exercise training enhances neuropsychological test performance, including semantic processing, in amnestic MCI patients, and these effects appeared to be stronger in women.

Summarizing the effects of exercise training in older adults diagnosed with MCI, significant effects tend to be observed when the exercise intervention involves moderate to high intensity exercise that is performed more than two days per week (*i.e.*, Baker *et al*. [73] and Lautenschlager *et al*. [71]). However, the differences between the exercise and control treatments do not necessarily reflect enhanced cognition or memory, but rather suggest maintenance of cognitive function occurred after exercise training relative to continued cognitive decline in the control condition. Another consideration is that the effects of exercise interventions in MCI patients have not been directly compared to healthy control groups. Thus, it is not clear if MCI patients benefit more, or less, from an intervention compared to those without this AD risk factor. 

One consideration regarding the variability in the outcomes observed in these exercise intervention studies is that common diagnostic criteria for MCI were not always clearly employed. As described by Petersen and colleagues [74], differences in the definition of mild cognitive impairment may explain variability among studies regarding the risk this diagnosis poses for future cognitive decline and dementia. The use of self-reported memory problems as the only indicator of MCI (e.g., Lautenschlager *et al*. [71]) without consideration of other domains of cognitive function and activities of daily living may result in a very heterogeneous sample that has greater cognitive deficits, or perhaps fewer, than a sample that does meet standard criteria. While the Petersen criteria have been widely cited, the diagnostic criteria for MCI are not without controversy (e.g., [75,76,77]), and there has been a recent recommendation that places MCI along a spectrum of pre-clinical to clinical symptom presentation coupled with biomarkers for AD-related pathology [41,78]. Diagnostic criteria notwithstanding, stable cognitive function over time is a desirable outcome even among healthy older adults, so the maintenance of cognitive outcomes after an exercise intervention in those with early signs of mild memory loss may be quite meaningful for these individuals. Optimally, exercise training in MCI would provide enough short-term protection to delay dementia onset. Nevertheless, it has yet to be demonstrated that exercise training in older adults diagnosed with MCI results in a long-term attenuation of cognitive decline, a delay in AD diagnosis, or a lesser AD conversion rate. 

## 5. Exercise Training with Increased Metabolic Risk for AD

Other than genetic and MCI risk factors, it is clear that metabolic disease processes also pose a risk for AD [79]. In a separate investigation, Baker and colleagues reported the results of their exercise intervention and stretching control conditions among cognitively intact individuals diagnosed with impaired glucose tolerance (*n* = 22) or just diagnosed Type 2 diabetes (*n* = 6) mellitus [80]. They found that performance was significantly better after six months on the Trail Making Test, Part B, during a task-switching task, and on the interference trials of the Stroop Color-Word Interference Test in the exercise group compared to the control group. Inspection of their data reveals, similar to the effects in MCI, that these effects were due as much to worsening performance in the control group as to improved performance in the experimental group. Among the metabolic markers, insulin sensitivity improved in the exercise intervention group compared to the control condition, and in both the exercise and control group, percent body fat and triglyceride levels were reduced. Other serum markers (β-amyloid 1–42, cortisol, BDNF, and IGF-1) did not significantly change after either condition. Again, the sample size was small; there were only nine patients in the control condition and included only one man, so sex differences could not be examined. Despite these shortcomings, this study is among the first to show improved cognition in older adults at increased metabolic risk for AD.

## 6. Physical Activity and Brain Structure

There have been several studies that have examined the effects of PA participation and exercise training on magnetic resonance imaging (MRI) derived measures of brain structure, such as regional tissue volume, regional cerebral blood volume, and indices of white matter structural integrity (for recent review see [65]). These studies, however, have included only very healthy older adults, have not assessed AD risk factors, or have specifically excluded individuals at increased risk for cognitive decline or AD. In fact, there are no published reports on the associations between PA or cardiorespiratory fitness and measures of brain structure in samples restricted to cognitively intact older adults who also vary on an established risk factor for AD. However, cross-sectional associations between cardiorespiratory fitness and brain volume, with consideration of APOE allele status, have been examined in samples of early-stage AD patients compared to healthy older adult controls. Based on the recently recommended diagnostic criteria for MCI and AD [41,42], some of these early-stage AD patients with CDR scores of 0.5 may be clinically indistinguishable from patients now described as “MCI due to AD” [76]. Given the paucity of data regarding interactions between PA and AD risk on measures of brain structure, we have included the few published reports in this area. 

Burns and colleagues [81] conducted graded exercise tests to quantify cardiorespiratory fitness (VO_2peak_) and collected MRI scans on 57 patients diagnosed with early-stage AD (CDR 0.5 to 1.0, mean age 74.3 years) and 64 healthy older adults without dementia (CDR = 0, mean age 72.7 years). Greater levels of cardiorespiratory fitness were associated with greater whole brain volume in the early-stage AD patients (*r* = 0.54, age-adjusted *b* = 0.35), but not in the healthy adults (*r* = 0.18, age-adjusted *b* = −0.20). Among the neurocognitive variables, there were significant relationships between fitness and Trail Making Test, Part B and Digit Symbol performance in the early-AD patients, but these associations did not maintain significance after adjustment for age. The lack of relationships between fitness and MRI and neuropsychological outcomes in the healthy controls are consistent with the associations reported by Etnier *et al*. [49]; however, Burns *et al*. [81] did not consider APOE genotype. 

Honea *et al*. [82] did consider APOE genotype in their follow-up study of correlations between VO_2peak_ and volume of specific brain regions using voxel-based morphometry in 71 patients diagnosed with early-stage AD (CDR 0.5 to 1.0; mean age 74.3 years; APOE-ε4 allele status: *n* = 7 homozygotes, *n* = 36 heterozygotes, *n* = 18 non-carriers) and 67 healthy older adults without dementia (CDR = 0; mean age 73.3 years; APOE-ε4 allele status: *n* = 3 homozygotes, *n* = 15 heterozygotes, *n* = 29 non-carriers), most of whom also participated in the Burns *et al*. [81] study. There were no relationships between fitness and regional gray or white matter volumes in the non-demented group. Within the early-AD group, greater fitness was associated with greater gray matter volume bilaterally in the inferior parietal cortex and the left parahippocampal gyrus, and showed trends (uncorrected for family-wise error) for positive associations in several other gray matter regions. In white matter regions, greater fitness in early-AD patients was associated with greater white matter volume in bilateral postcentral gyri and the left hippocampus. There were no significant interactions between fitness and APOE genotype in either the early-AD or healthy control groups. However, some lingering questions remain about this study. For example, because the associations with APOE genotype were analyzed separately within the patient and healthy control groups, this resulted in substantially unequal numbers of ε4 carriers *versus* non-carriers in each analysis. Thus, the two tests for the effect of APOE genotype may not have been optimized due to the smaller numbers of subjects in each analysis, and potential bias due to the unequal distribution of APOE genotype. A more balanced analysis with the data collapsed across groups (e.g., 51 heterozygotes *vs.* 47 non-carriers) would have provided greater statistical power and perhaps better addressed the question regarding interactions between fitness and APOE genotype. 

In a third study from the Burns group [83], change in cardiorespiratory fitness over a two-year period was assessed and correlated with cognitive and MRI outcomes. Patients diagnosed with early-stage AD (*n* = 37) were compared to healthy older adults (*n* = 53). A similar decline in fitness was observed in both groups over two-years, although fitness was lower at both time points in the early-AD group. Among the patient group, a greater decline in cardiorespiratory fitness was significantly associated with greater two-year atrophy in the left parahippocampal gyrus, and there were non-significant trends in the same direction for several other regions (insular cortex, right lingual gyrus, right inferior temporal gyrus, and right putamen). There were no significant associations between reduced fitness and brain atrophy in non-demented subjects. The similar rates of decline in fitness in both groups over two years suggest that AD pathology does not differentially affect aerobic capacity. The overall lower levels of fitness in the early-AD group, however, could reflect reduced PA due to cognitive decline, which cannot be determined without prospective data on PA behavior prior to diagnosis of AD. One cannot accurately determine the associations between AD and fitness without considering the potential impact of disease progression on PA behavior. Thus, caution is warranted in the interpretation of relationships between fitness and cognitive or MRI outcomes, and should not be interpreted as a reflection of exercise training or PA behavior *per se*. 

While several studies have reported the associations between fitness and PA on brain tissue volume, few have examined other MRI measures of brain structure that are altered in AD. Brain amyloid plaques, one of the major neuropathological markers of AD, now can be imaged using positron emission topography after injection of a radioactive tracer that binds to brain amyloid. Head and colleagues [84] recently reported the interaction between PA status and APOE genotype on cortical amyloid load using PET and the [^11^C] Pittsburgh Compound B (PiB). Cognitively intact older adults with a family history of AD (*n* = 163) were asked in a telephone interview about their walking, jogging and running activities over the past 10 years, which were converted to metabolic equivalent hours (MET-hours) per week. Those who met or exceeded the current recommendation of 7.5 MET-hours per week of PA were considered to have habitually high exercise levels, and those who did not meet this level were considered to have habitually low exercise levels. Among the sample, 52 participants were determined to be APOE-ε4 allele carriers (*n* = 39 low-exercise; *n* = 13 high-exercise) and 111 (*n* = 86 low-exercise; *n* = 25 high-exercise) were non-carriers. After controlling for age, education, BMI, and presence of hypertension, they found a significant exercise group by APOE status interaction. There was a greater effect of exercise engagement on the amyloid mean cortical binding potential in the APOE-ε4 allele carriers, with significantly lower brain amyloid in the high-exercise compared to the low-exercise group, an effect not observed in the APOE-ε4 non-carriers. Interestingly, this gene-behavior interaction was specific to cortical estimates of amyloid plaque, and was not significant for CSF Aβ42; although there were significantly higher CSF Aβ42 levels overall in the high-exercise groups (presumably reflecting greater brain amyloid clearance into CSF).

In summary, there is little known regarding the moderating effects of increased AD risk on the associations between PA, cardiorespiratory fitness, or exercise training on regional or whole brain volume. In healthy younger and older adults, being physically active and physically fit is associated with larger gray matter volume in several brain regions, and exercise training may produce angiogenic and neurogenic effects that can lead to anterior hippocampal growth in older adults [65]. Greater cardiorespiratory fitness is associated with greater brain volume in patients diagnosed with early-stage AD, however it is unclear if this advantage leads to better clinical outcomes for these patients. There are no published reports of the associations between PA or exercise training and brain volume among cognitively intact older adults at increased risk for AD. Finally, recent evidence indicates engaging in regular exercise at or above the minimum recommended levels may help to reduce amyloid burden in APOE-ε4 carriers more so than in non-carriers. However, prospective studies have not yet been conducted to confirm this association.

## 7. Physical Activity and Brain Function

A presumption of the neurotrophic effects of exercise is that these effects will translate into improved brain function, help preserve or maintain cognitive performance with age, and contribute to a reduction in incident dementia. When applied to an older adult population at increased risk for dementia, this assumption is tenuous at best due to the paucity of data bearing on this question.

Deeny and colleagues [85] examined the interaction between APOE-ε4 status and PA status on behavioral and cortical responses during the Sternberg working memory task among cognitively normal older adults ages 50–70 years (mean age 59.9 years). There was a behavioral testing portion of the study (*n* = 54, consisting of 16 APOE-ε4 carriers and 38 non-carriers), during which the Sternberg working memory task was administered and behavioral responses were recorded (reaction time and accuracy). A second session (*n* = 23, consisting of nine APOE-ε4 carriers and 14 non-carriers) involved magnetoencephalographic (MEG) recordings during performance of a modified version of the Sternberg task. For the behavioral session, PA energy expenditure was measured by self-report using the Yale Physical Activity Survey for Older Adults and was expressed as a continuous variable in kilocalories per week. A significant interaction between APOE-ε4 status and PA energy expenditure was found for reaction time. Among the APOE-ε4 carriers, greater PA energy expenditure was associated with faster reaction time, and this association was not observed in non-carriers. There was no interaction between APOE-ε4 status and PA for task accuracy, which was best explained by level of education. 

For the MEG portion of the Deeny *et al*. [85] study, PA status was dichotomized into a high PA group, defined by at least 3 days per week of aerobic exercise (*n* = 14; consisting of five APOE-ε4 carriers and nine non-carriers), and a low PA group, defined by less than 3 days per week of aerobic exercise (*n* = 9; consisting of four APOE-ε4 carriers and five non-carriers). The 160 channels of MEG data were averaged into eight regional montages. The right temporal region was the only region that showed a significant interaction between APOE-ε4 status and PA status. Among the APOE-ε4 carriers, those in the low PA group showed a lesser amplitude response (0 to 600 ms after probe onset) compared to both the high PA ε4 carriers and the low PA non-carriers. A further, exploratory, analysis into the temporal sequence of the cortical responses showed that the amplitude of the M170 component in the left and right temporal lobes was larger among high physically active participants regardless of APOE-ε4 status compared to the less physically active participants. For M170 latency, ε4 carriers showed longer latency of the response on average, but there were no significant interactions with PA status. A spatial localization analysis was conducted on a single subject (high PA non-carrier), which indicated the right fusiform gyrus as a possible source of the scalp recordings. Thus, the interaction between genetic risk and PA was observed for the behavioral reaction time data during the task in a larger sample of participants, but this interaction was not observed for the cortical responses. It is possible that the small number of participants in the MEG portion of the study contributed to the lack of an interaction effect, at least for the M170 latency variable.

In a more recent study, Deeny *et al*. examined temporal lobe glucose metabolism measured by FDG-PET and its associations with APOE genotype and cardiorespiratory fitness [86]. Fitness was assessed with a graded maximal exercise test in 18 women (mean age 62 years), half who carried at least one APOE-ε4 allele (ε2 allele carriers were excluded). Using unspecified criteria, two cardiorespiratory fitness groups of unspecified size, low fitness and high fitness, were created within each APOE genotype group. Within each APOE genotype group, the slope of the line between the low fit and high fit group was used as a predictor of brain glucose uptake in a linear regression analysis. Brain glucose uptake was measured on two different days after (1) quiet rest with eyes closed; and (2) performance of the Sternberg working memory task. The prediction of glucose metabolism based on their fitness metric was computed separately for ε4 allele carriers and non-carriers. Using this approach, they found that fitness did not predict resting glucose metabolism in the ε4 allele carrier or the non-carrier groups. However, when brain glucose metabolism was measured after the Sternberg task (during which the accuracy of both ε4 carriers and non-carriers was high (95%) and did not differ), significant associations with fitness were observed in the ε4 carriers, but not in the non-carriers. Then, in a further *post-hoc* comparison analysis, the difference in glucose metabolism between the high fit and low fit ε4 carriers was analyzed. Among the ε4 carriers, greater fitness was associated with greater glucose uptake in the left inferior temporal cortex, a finding in the opposite hemisphere in comparison to the previous MEG study that used the same task [85]. Lower fitness among ε4 carriers was associated with greater glucose uptake in several regions, including bilateral middle frontal gyrus, right superior frontal gyrus, right inferior parietal gyrus, and left postcentral gyrus. A few considerations regarding this study are the lack of clarity regarding the fitness metric that was employed, the small sample, and that the ε4 allele carriers were more educated than the non-carriers (19.0 *versus* 17.2 years, a difference of effect size *d* = 0.76). While education level was reported, education was not accounted for in any of the analyses, so it is not clear the effects are independent of (or could possibly be explained by) differences in education between the groups. 

Functional magnetic resonance imaging (fMRI), which has the advantage of considerably greater spatial resolution compared to MEG (though considerably less temporal resolution), can be measured during the performance of a memory task (unlike FDG-PET), and has been used as a biomarker to longitudinally track and predict future cognitive decline [7]. Given that episodic memory impairment is the core deficit of AD [87], most fMRI studies of AD risk have used episodic memory tasks [88]. However, episodic memory impairment, which can be documented without neuroimaging, is not restricted to patients diagnosed with MCI or AD [38,74,89,90,91] but has also been observed in normal aging [92]. Individuals who are in the preliminary stages of cognitive decline may exert greater effort during episodic memory tasks and may paradoxically display a greater BOLD signal [93] due to the increased cognitive challenge, thus confounding the results. Indeed, in longitudinal studies, participants who display a greater extent of activation during episodic memory tasks are more likely to show future cognitive decline [94,95,96]. The key to understanding the extent to which undetected neuropathology in cognitively intact but genetically at risk individuals may influence fMRI measured brain hemodynamics during memory retrieval is to utilize a memory retrieval task that requires little effort and that can be performed with a high level of accuracy even among those with mild episodic memory impairments. In doing so, these neuroimaging data may become useful in predicting future cognitive decline, or as a marker of responsiveness to an intervention such as exercise.

Unlike episodic memory skills, semantic memory abilities (e.g., recall of general facts and knowledge about the world that is not contextually specific) remain relatively intact with normal aging [92] but are vulnerable to the earliest stages of AD [97,98,99,100,101]. Contrary to episodic memory tasks [94,95,96], participants who exhibit *lower* activation during semantic tasks may be at the greatest risk of future cognitive decline [7,102]. Furthermore, the semantic memory system overlaps with regions associated with the default mode network [103] and brain areas that are susceptible to AD neuropathology [104]. Thus, analysis of the BOLD signal during semantic memory processing has a number of advantages over episodic memory for discriminating those at increased risk for AD [105], and may provide a useful biomarker for response to an intervention.

Two studies recently examined the associations between PA and semantic memory-related brain activation. In both studies, brain activation was measured using functional magnetic resonance imaging while participants performed a famous name discrimination task. In this task, the participant makes a right index finger button press to indicate the name is famous (e.g., “Frank Sinatra”) and a right middle finger button press to indicate the name is not famous (e.g., “Rebecca Hall”). Older adults, even those with cognitive impairment, perform the task at about 90% accuracy. Only correct trials are included in the analysis in order to remove activation related to errors in memory performance. In the analysis of the brain activation response, a ‘famous’ minus ‘unfamiliar’ metric is calculated in order to remove brain activation related to the common sensory and motor aspects of the two name conditions. Importantly, in cognitively normal older adults, we have found that the degree of semantic memory activation during this task is predictive of who will show evidence of cognitive decline after a period of 18 months, with greater activation (*i.e.*, more red in the brain maps) related to greater cognitive stability over time [7] (see [106] for a review). 

**Figure 1:**
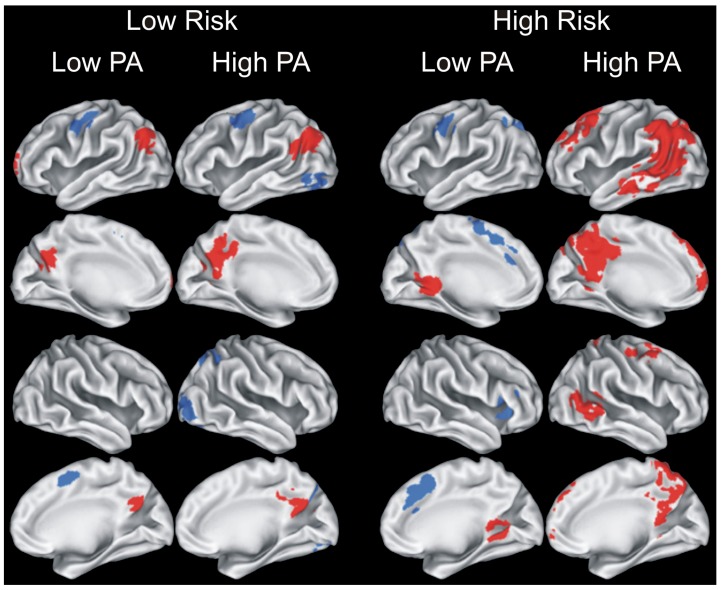
Results of voxelwise analysis by Smith *et al*., 2011 [107] showing brain regions with significant differences between Famous and Unfamiliar name conditions for each of the four groups (Low Risk/Low PA; Low Risk/High PA; High Risk/Low PA; and High Risk/High PA). Areas in red indicate Famous > Unfamiliar; blue areas indicate Unfamiliar > Famous. PA = physical activity. Figure reproduced with permission from [107], Copyright © 2011, Elsevier.

**Figure 2:**
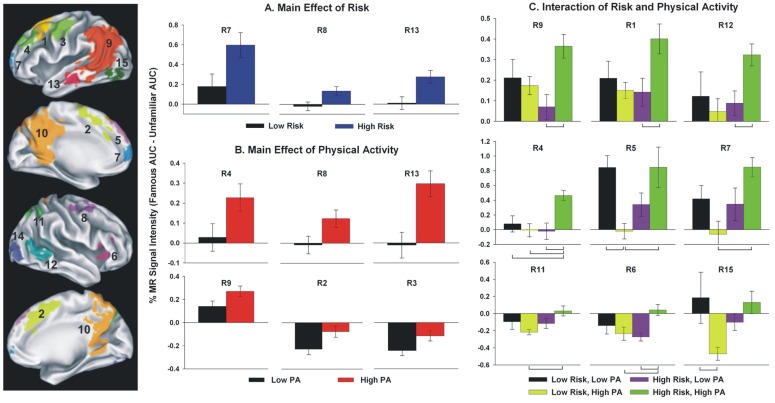
Fifteen functional regions of interest (fROIs) identified by Smith *et al*., 2011 [107]. In the left panel, region numbers (R#) correspond with activation foci shown on the right. Bar graphs represent mean percent MR signal intensity change for main effects of Physical Activity and Risk (panels **A** and **B**, respectively) and interaction effect of Physical Activity × Risk (panel **C**). *Post-hoc* group differences are indicated by brackets in panel **C** (*p* < 0.01). Error bars = S.E.M. Figure reproduced with permission from [107], Copyright © 2011, Elsevier.

Greater levels of PA have been associated with greater brain activation during the famous name discrimination task, particularly in those at increased genetic risk for AD. Smith and colleagues [107] obtained MRI data in four groups of healthy older adults (*n* = 17 each) who varied in PA and genetic risk: (1) Low Risk/Low PA; (2) Low Risk/High PA; (3) High Risk/Low PA; and (4) High Risk/High PA. Physical activity level was based on self-reported frequency and intensity using the Stanford Brief Activity Survey [108,109]. AD risk was based on presence or absence of the APOE-ε4 allele. A significant interaction between PA status and genetic risk was found, with the High PA/High Risk group demonstrating greater semantic memory activation in several brain regions compared to the remaining three groups (see Figure 1, right; Figure 2). Importantly, these groups did not differ in age, education, cognitive status, depression, or on a battery of neuropsychological tests of episodic memory and executive function. Taken together with the finding that greater semantic memory related activation during fame discrimination predicted cognitive stability over 18-months [7], this pattern suggests that increased levels of PA may provide neuroprotection and could delay cognitive decline, especially among APOE-ε4 allele carriers. 

In a recent study, Woodard and colleagues [10] compared engagement in cognitively stimulating activities *versus* PA in the longitudinal prediction of future cognitive decline. Seventy-eight cognitively intact older adults (*n* = 26 APOE-ε4 allele carriers; *n* = 42 non-carriers) completed baseline and 18-month neuropsychological assessment, a structural MRI, and task-activated fMRI during the famous name discrimination task. At the baseline assessment, all 78 participants were cognitively intact. At the 18-month follow-up, 27 participants were determined to have declined by at least one standard deviation on neuropsychological testing. The stable and declining groups did not differ at baseline in age, education, sex, ethnicity, family history of dementia, cognitive activity scores, or PA. The groups did differ, however in the proportion of APOE-ε4 allele carriers (12/51 in the stable group *versus* 14/27 in the declining group). In addition to APOE allele status, the baseline measurements of hippocampal activation, hippocampal volume, and self-reported engagement in cognitively stimulating activities and PA were entered into logistic regression prediction models to determine which baseline factors best-predicted future cognitive decline. Cognitive activity, alone or in combination with hippocampal activation or hippocampal volume, did not predict the probability of future cognitive decline. Physical activity, on the other hand, significantly reduced the probability of future cognitive decline, but only in APOE-ε4 allele carriers. The protective effect of PA in APOE-ε4 allele carriers was shown to be consistent when both hippocampal volume and hippocampal activation were held constant, although the interaction was no longer significant when either hippocampal activation or hippocampal volume were extremely high or extremely low. These data indicate that PA provides protection against cognitive decline in APOE-ε4 allele carriers. However, this protective effect may not be as potent when the individual already has robust hippocampal volume or hippocampal function, possibly indicating existing neural or cognitive reserve [110,111], or on the other hand, when hippocampal atrophy or inability to activate the hippocampus during memory retrieval has occurred, possibly indicating PA cannot rescue individuals when substantial hippocampal impairment exists. The caveat to this view, however, is that the directions of causality between baseline PA status and baseline hippocampal structure and function are not known. It may be the case that previous (unmeasured) PA contributed to baseline hippocampal reserve; likewise, prior hippocampal atrophy may have contributed to reduced PA behavior measured at baseline.

**Figure 3:**
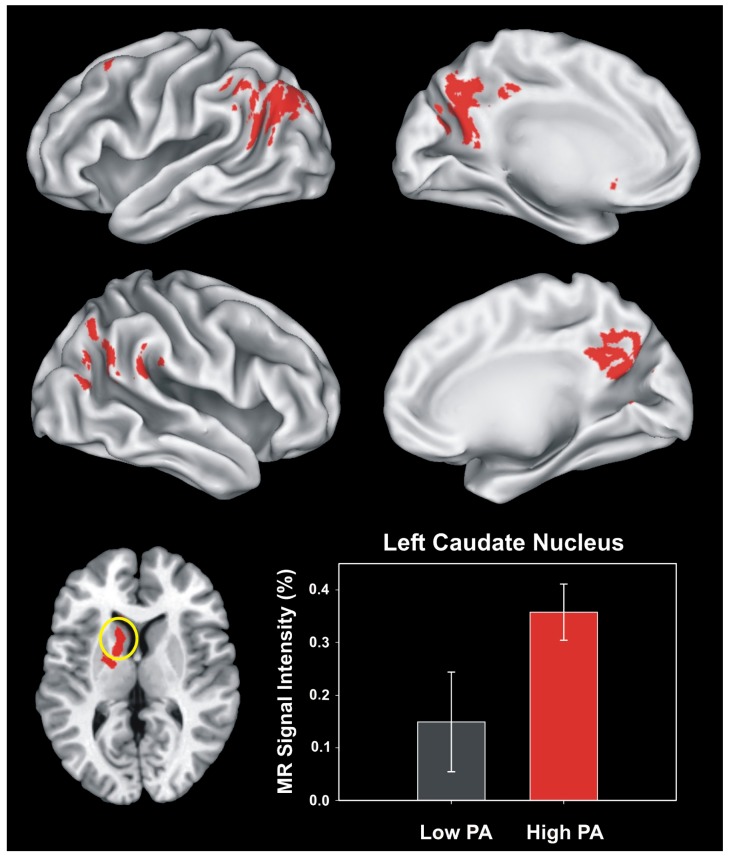
Functional Regions of Interest (fROIs) derived from all regions activated in both MCI groups in Smith *et al*., 2011 [112]. The six regions of interest are shown in the top (left hemisphere) and middle (right hemisphere) panels. The two groups significantly differed only in activation (Famous > Unfamiliar) of the left caudate region (circled in yellow, axial view in the lower left panel, *z* = +10 mm superior to the AC-PC line). The MR signal intensity difference (%AUC) between the Low-PA and High-PA groups in the left caudate region is shown in the lower right panel. Error bars = SEM. Figure reproduced with permission from [112], Copyright © 2011, Elsevier.

In a study of MCI patients, Smith and colleagues [112] examined associations between self-reported PA and fMRI activation during the famous name discrimination task. The MCI patient groups did not differ in age, education, sex, neurocognitive test performance, or APOE allele status. The patients were classified as physically inactive (Low PA) or physically active (High PA) based on their self-report on the Stanford Brief Activity Survey [109], and underwent an fMRI scan while they performed the famous name discrimination task. Performance on the task did not differ between the groups, but greater activation of the left caudate nucleus occurred during fame discrimination in the more physically active MCI patients (see Figure 3). Although the caudate is not viewed as a primary component of memory circuits, changes in caudate volume are pronounced in the progression of dementia and may be affected by AD pathology [113,114]. The caudate contributes indirectly to cognitive processing through the augmentation of intentional actions and cognitions, and the inhibition of competing motor programs and cognitive networks through closed loops that are modulated by dopamine D1 receptor activity. D1 receptor activation is hypothesized to increase the signal-to-noise ratio on the outputs of striatal spiny neurons and may facilitate cognition by helping to prolong the activation of the cortical loops that support them [115]. The finding by Smith and colleagues [112] suggests that being physically active may help preserve activation of the caudate during semantic memory retrieval in MCI patients. Exercise is known to increase brain dopamine levels and enhance D1 receptor function [116]. In MCI patients, it is possible that PA may increase dopaminergic input and augment the gain in frontostriatal closed loop networks, leading to enhanced activation of the caudate during semantic memory retrieval [115]. 

An important interpretive question is whether enhanced semantic memory processing represents a positive or a negative indicator of future cognitive decline? In a longitudinal study of initially cognitively intact individuals, greater baseline cortical and hippocampal fMRI activation during the famous name recognition task was protective against a decline in neurocognitive performance after 18 months, independent of APOE allele status [7]. That is, lesser cortical and hippocampal fMRI activation at baseline, measured prior to knowledge regarding who would show future cognitive change, accurately predicted cognitive decline 18 months later. The study by Woodard *et al*. (2012) [10] further demonstrated that the protective effect of PA, with greater PA associated with cognitive stability over 18-months, was specific to APOE-ε4 allele carriers. Taken together, the results from these three studies suggest that PA is associated with enhanced memory circuit activation in cognitively intact individuals at increased genetic risk for AD in a manner that is protective against future cognitive decline. Furthermore, it is possible that these effects are specific to initially cognitively intact APOE-ε4 carriers who have, for whatever reason, neither extremely large nor extremely small hippocampi.

## 8. Potential Mechanisms for Physical Activity Benefits to Those at Greater Risk for Alzheimer’s Disease

Volitional exertion has pleiotropic effects that are very difficult to disentangle. There are several candidate mechanisms that have been put forth (for review see [117]). For example, in rodent models, exercise has been well documented to stimulate neurotrophic effects (e.g., brain derived neurotrophic factor, insulin-like growth factor-1) and exercise leads to neurogenesis in the dentate gyrus [118,119]. In addition, the cholinergic effects of exercise may increase perfusion, enhance neural recruitment, and possibly attenuate the accumulation of beta-amyloid in rodents [84,120]. There is also preliminary evidence that exercise may have these neurotrophic effects in the hippocampal formation in healthy adults [16] and healthy older adults [23]. However, whether these potential mechanisms are more potent among APOE-ε4 carriers or others at increased AD risk is unknown.

A major consequence of possession of the APOE-ε4 allele is a disruption of lipid homeostasis, which has negative consequences on amyloid precursor protein (APP) function and the handling of brain amyloid, as well as on cholinergic function and neuroinflammation [121,122]. Apoliproproteins are lipid carrier molecules essential to regulating the metabolism of lipid in response to neuronal injury. The protein apolipoprotein E (apoE) in particular is highly involved in brain neuronal growth and repair through its role in regulating cholesterol and phospholipids associated with synaptic plasticity [121]. Because lipoproteins associated with the ε4 allele are cleared more easily, the amount of apoE available in the brain (produced mainly by astrocytes) is greatly diminished compared to non-ε4 carriers [123]. This also results in a reduction of lipoprotein lipase activity leading to decreased availability of free fatty acids (FFA) to brain cells, which are essential elements of neuronal repair and neurotrophic processes [122]. It has been hypothesized that this cycle causes alterations in APP function that promote the formation and retention of brain β-amyloid [121]. Altered lipid membrane homeostasis also reduces glycolytic metabolic processes in a manner that reduces the availability of acetyl-CoA-derived ATP and acetylcholine (Ach) [122,123], leading to a deficit in cholinergic function. Exercise training enhances cholinergic function, and has been shown to reverse the activation of acetylcholine esterase (AChE) in the hippocampus and cerebral cortex of rats [124]. 

There are a few animal studies that have described effects of exercise on brain function that interact with APOE genotype. In a transgenic mouse model, APOE-ε4 mice showed a comparable increase in hippocampal BDNF *versus* APOE-ε3 mice after both completed 6 weeks of voluntary wheel running. Furthermore, tyrosine kinase B receptors, which have a high affinity for BDNF, were increased in APOE-ε4 to the level of APOE-ε3 mice after wheel running [18]. There is also evidence that exercise training in apoE deficient mice may rescue the progression of peripheral atherosclerotic processes, effects that appear to be mediated through exercise-induced increases anti-inflammatory cytokines [125]. Unfortunately, little is known about the effects of exercise training on human brain lipid homeostasis in ε4 carriers [126], and whether or not responses to exercise training differ based on APOE genotype has not been clearly established [127]. What we do know in humans is based on measures from peripheral blood. For example, exercise training increased the particle size and cholesterol concentration of high density lipoproteins [128], though to a greater extent in ε2 carriers [129], and decreased the diameter of very low density lipoproteins in both ε4-carriers and non-carriers [130], both of which are cardio-protective effects. 

Taken together with the evidence in humans, it appears that the effects of APOE-ε4 genotype on AD-related neuropathology and its clinical manifestation of memory impairment are exacerbated by physical *inactivity*. Indeed, PA does not have greater effects in ε4 carriers when compared to non-carriers, but rather these associations tend to be greater in physically active ε4 carriers compared to sedentary ε4 carriers. Thus, one may speculate that exercise and PA may produce counteractive effects of the phenotypic expression of the APOE-ε4 allele, possibly through benefits to brain lipid metabolism, neuroinflammation, and cholinergic function [117]. In future animal research, it will be important to test hypotheses regarding potential neurophysiological mechanisms of exercise that are specific to APOE genotype. These studies will help not only to elucidate the benefits of exercise for brain health, but also to inform the development of pharmacologic compounds for AD treatment and prevention. 

## 9. Recommendations for Future Research

Additional longitudinal epidemiologic studies to examine cross-sectional associations between PA and/or fitness on cognitive outcomes are needed. In particular, there is a need to prospectively assess the impact of changes in PA over time in relation to cognitive trajectory and brain function, and whether or not increases or decreases in PA behavior differentially impact those a greater risk for AD compared to those with normal risk. For example, it is not known if a decrease in PA from previously high levels would accelerate cognitive decline, or if the past history of regular PA is protective, and whether these effects would depend on genetic risk or on cognitive status at the time of the change in PA. There is also a need for smaller laboratory based interventions, focused on answering mechanistic questions related to brain function, to help guide the design of large-scale randomized clinical trials. Prospective exercise training interventions in those at increased risk for cognitive decline, with measures of cognition and memory function using standardized neuropsychological tests and multi-modal MRI outcomes are necessary to demonstrate the efficacy for exercise interventions to prevent AD in those at increased risk, or as adjuncts to treatment for AD patients. Moreover, PA and exercise interventions should be examined in conjunction with, and in comparison to, other pharmacologic and non-pharmacologic interventions. These interventions should be designed in partnership with community-based organizations to facilitate the translation and implementation of these interventions in community-based settings. Finally, a critical issue is to characterize potential mechanisms *in humans* that may underlie the observed neuroprotective effects of exercise and PA. EEG studies aside (none of which have considered AD risk status; see review by [131]), the available neuroimaging evidence in humans is based primarily on MRI or PET scans obtained on a day that exercise was not performed. The studies reviewed here have examined the longer-term effects of exercise training on, or associations of PA behavior with, brain function or structure in older adults. However, these studies, which are clearly important, have not addressed fundamental questions regarding how a single session of exercise may impact older adult brain function, and if these effects differ based on the possession of established risk factors for AD. An understanding of the acute effects of exercise is important, as the neural adaptations that occur in response to repeated single bouts of exercise are the presumed building blocks for long-term neuroprotective adaptations. 

## 10. Summary and Conclusions

Leisure-time PA and exercise training are known to help maintain cognitive function in healthy older adults. However, relatively little is known about the effects of PA on cognitive function or brain function in those at increased risk for AD. In large epidemiological studies, the results have been inconsistent. Some have shown that the protective effects of PA on risk for future dementia appear to be larger in those at increased genetic risk for AD, but others have not observed this association. Exercise training is also effective at helping to promote stable cognitive function in MCI patients, and greater cardiorespiratory fitness is associated with greater brain volume in early-stage AD patients. Greater levels of PA also may reduce the likelihood of amyloid deposition in APOE-ε4 allele carriers more so than in non-carriers, and may promote the activation of semantic memory-related neural circuits to a greater extent in those at increased genetic risk for AD. A greater research emphasis should be placed on randomized clinical trials for exercise, with clinical, behavioral, and neuroimaging outcomes in people at increased risk for AD. Longitudinal community-based primary prevention trials are needed to determine if exercise or PA programs, in comparison to standard treatment or prevention approaches, may affect brain function and cognition, and ultimately reduce the incidence of AD and other dementias.
